# Human Papillomavirus Oral Infection: Review of Methodological Aspects and Epidemiology

**DOI:** 10.3390/pathogens10111411

**Published:** 2021-10-30

**Authors:** Eugenia Giuliani, Francesca Rollo, Maria Gabriella Donà, Anna Rosa Garbuglia

**Affiliations:** 1 Scientific Direction, San Gallicano Dermatological Institute IRCCS, Via Elio Chianesi 53, 00144 Rome, Italy; eugenia.giuliani@ifo.gov.it; 2 Pathology Department, Regina Elena National Cancer Institute IRCCS, Via Elio Chianesi 53, 00144 Rome, Italy; francesca.rollo@ifo.gov.it; 3 STI/HIV Unit, San Gallicano Dermatological Institute IRCCS, Via Elio Chianesi 53, 00144 Rome, Italy; 4 Laboratory of Virology, National Institute for Infectious Diseases, INMI Lazzaro Spallanzani IRCCS, Via Portuense 292, 00149 Rome, Italy; argarbuglia@iol.it

**Keywords:** Human Papillomavirus, Alphapapillomavirus, Betapapillomavirus, Gammapapillomavirus, detection method, oral infection, oropharyngeal infection, prevalence, incidence, clearance

## Abstract

Oral infection by Human Papillomavirus (HPV) has recently gained great attention because of its involvement in the development of a subset of head and neck squamous cell carcinoma. The role of specific Alpha-HPVs in this regard has been well established, whereas the contribution of other genera is under investigation. Despite their traditional classification as “cutaneous” types, Beta and Gamma HPVs are frequently detected in oral samples. Due to the lack of a standardized protocol, a large variety of methodologies have been used for oral sample collection, DNA extraction, HPV detection and genotyping. Laboratory procedures influence the evaluation of oral HPV prevalence, which largely varies also according to the population characteristics, e.g., age, gender, sexual behavior, Human Immunodeficiency Virus (HIV) status. Nevertheless, oral infection by Beta and Gamma HPVs seems to be even more common than Alpha-HPVs. The latter is 5–7% in the general population, and increases up to 30% approximately in HIV-infected men who have sex with men. Despite major advances in the evaluation of oral HPV prevalence, its natural history is still little understood, especially for Beta and Gamma HPVs. The latest technologies, such as Next Generation Sequencing (NGS), can be exploited to gain new insights into oral HPV, and to improve the identification of novel HPV types.

## 1. Introduction

Human Papillomaviruses (HPVs) cause the most widespread sexually transmitted infection [1]. HPVs, which belong to the *Papillomaviridae* family, are small, non-enveloped viruses with a circular double-stranded DNA encapsulated within an icosahedral capsid. To date, around 220 HPV types have been completely sequenced, and there are numerous potential new types that remain unclassified. Based on variations in the sequence of the L1 open reading frame, which encodes for the major capsid protein, HPVs are classified into five genera (Alphapapillomavirus, Alpha-HPV; Betapapillomavirus, Beta-HPV; Gammapapillomavirus, Gamma-HPV; Mupapillomavirus, mu-HPV; Nupapillomavirus, nu-HPV) [2] but the majority of them belong to Alpha-HPV, Beta-HPV and Gamma-HPV genera.

Historically, HPVs have also been divided into two different groups based on their tropism for either cutaneous or mucosal epithelia. Thus, in the literature, the Alpha-HPVs are still referred to as “mucosal” and the Beta-HPVs and Gamma-HPVs are defined as “cutaneous”. However, the growing evidence that demonstrates “cutaneous” HPV broad distribution at mucosal sites [3,4,5,6,7] have led some scientists to reconsider the tropism-based nomenclature that has long been believed an incontrovertible paradigm [8].

Alpha-HPVs include Low-risk and High-risk types, as classified by the International Agency for Research on Cancer (IARC) based on their carcinogenic potential [9]. The High-risk types, i.e., HPV 16, 18, 31, 33, 35, 39, 45, 51, 52, 56, 58 and 59, have a distinctive oncogenic potential, being the aetiological agents of cervical, vulvar, vaginal, penile, and a subset of head and neck squamous cell carcinoma (HNSCC) [10]. In particular, HPV has a predominant role in the development of oropharyngeal squamous cell carcinoma (OPSCC) [11,12].

The contribution of HPV to the above-mentioned neoplasias ranges between 25% (vulvar carcinoma) and virtually 100% of cases (cervical and anal carcinomas) [10]. Particularly, HPV16 represents the most frequent type in all the HPV-associated cancers, and together with HPV18, it contributes approximately to 60–90% of cases [13,14,15,16,17]. Beta and Gamma genera include HPV types that infect the squamous stratified epithelium of the skin of the whole human body, so they are considered as a part of the healthy skin commensal flora [18]. Beta-HPVs are distributed on the forehead, back of the hand, the buttocks and the genital skin, where they reside in the hair follicle, which is considered their natural reservoir [19]. Nonetheless, the distribution of Beta and Gamma HPVs, as already mentioned, also includes non-cutaneous anatomical sites, such as the nasal mucosa [4], the oral cavity and the anal canal [6,20]. HPVs belonging to Gamma genus have been less extensively investigated, especially as far as concerns the associated diseases and their biological activities, which deserve more investigation [21].

Because of the causal association of this virus with HNSCC, current studies in the HPV field largely focus on oral infection. In this review, we will firstly discuss the most commonly used methods for oral sample collection, detection and genotyping of Alpha, Beta and Gamma HPVs. We will then outline the epidemiology of the infection caused by the three genera. Finally, a brief overview of the lesions caused by these HPV genera at the level of the head and neck region will be provided.

## 2. Oral Infection by Alpha, Beta and Gamma HPVs: The Methodological Aspects

**Figure 1:**
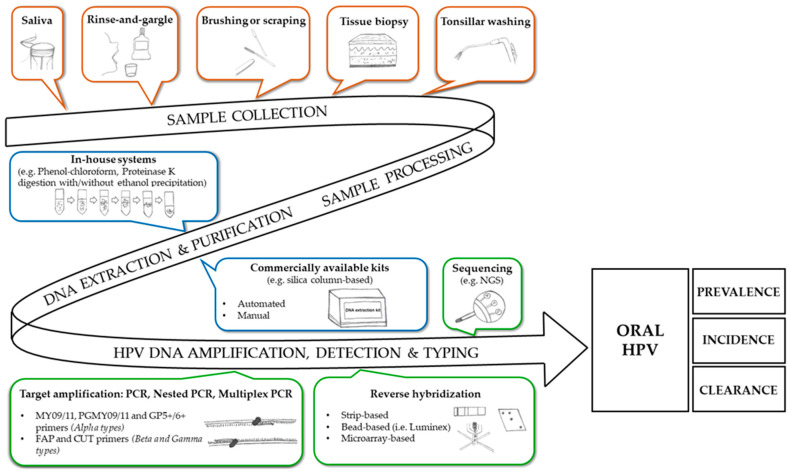
Laboratory workflow for the assessment of oral HPV infection. The operating laboratory procedures are represented in sequence: the oral sample collection (followed by sample processing), DNA extraction and purification, HPV-DNA amplification, detection and typing. Numerous HPV testing methods are available but only the methodologies mentioned in the text are depicted.

The laboratory workflow for the assessment of oral HPV infection is depicted in Figure 1. In brief, this includes the following steps: (1) oral sample collection (followed by a pre-analytical phase); (2) nucleic acid extraction and purification; (3) HPV-DNA amplification, detection and genotyping. The procedures related to these steps will be described later. However, due to the wide variety of the methods available, the following sections are not intended to be exhaustive.

### 2.1. Sample Collection, Nucleic Acid Extraction and Purification

So far, no gold standard for oral sample collection has been identified. Many kinds of samples have been used for oral HPV detection, the main being: saliva, oral rinse-and-gargle, tonsillar washing, mucosal scraping or brushings and tissue biopsies. It has been estimated that approximately 100,000 cells should be processed to obtain reliable results with Southern blot hybridization [22]. More sensitive methods for HPV DNA detection require a much lower input. Despite the fact that the amount of material obtainable through the different sample collection techniques may vary largely, all of them make it possible to recover enough material for very sensitive detection assays. In real-time PCR, for instance, DNA copy number equivalent to 10^3^ human diploid cells are generally used. A higher amount of human genomic DNA could negatively affect the sensitivity of HPV-DNA detection by inhibiting the amplification of the HPV target region [23,24].

Saliva samples are usually shaken and incubated at 50 °C–56 °C for 1–2 h before nucleic acid extraction [25,26,27,28]. Oral rinse-and-gargles are obtained with 10–15 mL of commercial mouthwashes, buffer saline or water, used for rinse and gargle cycles of 30–60 s. In the pre-analytic phase, these samples are centrifuged (at least at 2000 rpm for 10 min) to obtain the cell pellet. This is then washed, e.g., with 1× phosphate-buffered saline (PBS), and finally suspended in an appropriate buffer [29].

De Souza et al. [30] compared the sensitivity obtained with oral samples collected with 7 mL of saline (0.9%) vs. saliva samples, collected with the OMNIgene kit for microbial DNA (DNA Genotek, Ottawa, ON, Canada). Both samples (200 µL) were extracted using the same kit (Promega Maxwell viral kit, Madison, WI, USA), and HPV-DNA was amplified with two different PCR: (1) single round PCR with GP5+/6+ primers; (2) nested PCR using MY09/11 outer primers and GP5+/6+ inner primers. Using the oral rinses, HPV prevalence was 10.4% with GP5+/6+ PCR vs. 11.5% with nested PCR. For saliva samples, the respective prevalences were 3.1% vs. 16.7%. The agreement between matched oral rinses and saliva samples from the same individuals was satisfactory when analyzed with the same HPV detection method (88% with GP5+/6+ single round PCR, and 84% with nested PCR). The agreement was lower when the same sample was tested with the two different PCR detection methods (82%). The nested PCR was found to be more type-sensitive, detecting a wider range of HPV types, and a lower number of HPV-DNA copies in multiple infections than MY09/11 PCR or GP5+/6+single round PCR, as previously described for cervical samples [31]. 

Comparison of oral gargle and tonsillar washing (performed by a clinician using a specific device) was carried out by Choo et al. [32] to investigate whether the addition of tonsillar washing to gargling could improve HPV detection. Once resuspended in PBS, exfoliated cells from both types of sample were used for nucleic acid extraction with a QIAamp DNA Mini Kit (QIAGEN, Hilden, Germany). HPV-DNA was then detected using a nested PCR (PGMY09/11 primer set for the first round, GP5+/6+ primer set for the second round), followed by the direct sequencing of the purified PCR products on a genetic analyzer. The GENOSEARCH HPV31 assay, able to detect 31 HPV types (High-risk HPVs: 16, 18, 31, 33, 35, 39, 45, 51, 52, 56, 58, 59, 68; Low-risk HPVs 6, 11, 26, 42, 44, 53, 54, 55, 61, 62, 66, 70, 71, 73, 82, 84, 90, CP6108) was also utilized to test the same samples. This method is based on a multiplex PCR followed by a hybridization with genotype-specific probes on a Luminex platform. Using the nested PCR, it was observed that 64.7% of participants with a positive oral gargle were HPV-positive also in the corresponding tonsillar washing, while 1.0% of those with an HPV-negative oral gargle were HPV-positive in the corresponding tonsillar washing. Similar results were obtained with GENOSEARCH HPV31: 70.6% of samples were HPV-positive in both the oral gargle and tonsillar washing, while 1.4% of the patients were negative in the oral gargle, and positive in the respective tonsillar washing. Based on their results, the authors concluded that the addition of tonsillar washing only marginally facilitated HPV detection compared with the oral gargle alone. A higher HPV detection rate using oral rinse-and-gargles compared to oropharyngeal and oral brushings was also demonstrated in another study [33]. Comparing the HPV findings in matched samples analyzed with the same HPV detection method, a higher sensitivity of the oral rinse-and-gargle for the detection of high-risk HPVs and HPV16 was observed. The inability to brush the tonsillar crypts in vivo deeply enough to obtain exfoliated cells from the oropharynx may limit the utility of the oropharyngeal brushings.

Besides the wide spectrum of oral specimens that can be collected, and the variability of the pre-analytic phase, a large variety of methods are available for extraction and purification of nucleic acids. Notably, the extraction method can influence the sensitivity of HPV detection. D’Souza et al. [34] compared different methods of nucleic acid extraction from oral rinses: (1) phenol-chloroform; (2) Puregene (which includes pellet collection by centrifugation); (3) the QIAamp kit (QIAGEN, Hilden, Germany); (4) proteinase K digestion with and without (5) ethanol precipitation. Puregene purification resulted in the detection of the greatest number of HPV-positive subjects and HPV infections. The weakest sensitivity was observed when samples were treated only with proteinase K digestion, possibly because of the presence of PCR inhibitors in the oral rinse. PCR inhibition seems to be a more relevant issue for oral samples than samples from other anatomic sites that are susceptible to HPV infection. D’Souza et al. concluded that studies that utilize only ethanol precipitation or phenol-chloroform extraction could have underestimated HPV prevalence by 40 to 75%. These data underline the important role of DNA purification in avoiding the misclassification of HPV status (false negative results) in oral exfoliate samples and, more generally, how important it is to optimize all methodologies used in HPV-DNA detection.

### 2.2. HPV-DNA Detection and Typing

#### 2.2.1. Alpha-HPVs

The assays used to assess the presence of Alpha-HPVs in oral samples are primarily those validated/employed for the diagnostics of this infection in cervical and anal samples. Currently, more than 250 commercial tests are available for the molecular detection of Alpha-HPVs [35]. Some of them target the most important high-HPV types without distinguishing the individual genotypes. Others provide full (individual determination of the HPVs) or partial (differencing those with the highest oncogenic potential) genotyping information. HPV tests for the assessment of Alpha-HPV infection also differ according to the technology they rely on, e.g., real-time PCR-based tests or reverse hybridization-based tests. In most of the cases, primers targeting a conserved region of L1 gene are used for the amplification of HPV-DNA, and a nested PCR can be employed to reach a greater sensitivity. Indeed, a nested PCR is generally recommended to amplify genotypes that are present in low copy numbers. The most used set of primers comprises MY09 (5’ CGT CCM ARR GGA WAC TGA TC 3’) and MY11 (5’GCM CAG GGW CAT AAY AAT GG 3’) [36]. This can be used in the first round of a nested PCR, followed by GP5+ (5’ TTT GTT ACT GTG GTA GAT ACT AC 3’) and GP6+ (3’CTT ATACTA AAT GTC AAA TAA AAA G 5’) [37] for the second round. MY09/MY11 primers have been modified to obtain a PGMY09/11 set of primers, which consists of a pool of primers [38].

Moreover, broad range SPF10 primers [39,40] have been widely used to detect HPV in oral samples [41,42,43]. This primer set targets a 65 base pair fragment of L1 region and is commercially available in two different HPV tests: (i) the original SPF10-DNA enzyme immunoassay (DEIA)-LIPA25 system, where a DEIA performed in a 96-well format is followed by a reverse hybridization for 25 HPVs [43]; (ii) a reverse blotting hybridization system, which is based on hybridization of the biotinylated amplicons with probes immobilized on nitrocellulose strips and detects up to 32 HPV genotypes, depending on the kit version (INNO-LiPA HPV Genotyping, Innogenetics/Fujirebio, Göteborg, Sweden).

#### 2.2.2. Beta and Gamma HPVs

The methods available for the detection of Beta and Gamma-HPVs in oral samples can often identify only a restricted number of types. This may make it difficult to assess their actual prevalence in asymptomatic subjects and HNSCC.

Currently, there are different PCR-based methods that can be used for Beta and Gamma HPV-DNA detection in skin and oral samples [44]. These include the FAP primer system [45,46,47], which has been a successful approach in identifying many Beta and Gamma HPV types [48,49]. Other generic primers (CUT), initially designed to detect Beta and Gamma HPVs in skin samples [50], can also be used for oral samples. This mix of five primers [CUT1Fw 5’TRCCiG AYC CiA ATA ART TTG 3’; CUT1AFw (5’TRCCiG AYC CiA ACA GRTTTG3’), CUT1BFw (5’TRC CiG AYC CiA ATA GRT TTG3’), CUT1CFw (5’TRC CiG AYC CiA ACA AAR TTT G3’), CUT1BRv (5’TCi ACC ATR TCi CCR TCY T3’)] was selected starting from the sequence of 88 HPV types (Alpha, Beta and Gamma). The authors used the CUT primer system in a single tube “hanging droplet” PCR to improve the sensitivity for Beta and Gamma HPV detection and to minimize the risk of cross contamination. In this protocol, the reaction mixture for the second PCR round is placed inside the cap of the tube used for the first round. Once the first round is completed, the “hanging droplet” is incorporated into the reaction mixture by spinning down the tube [51]. The hanging droplet system can be applied to FAP nested PCR using FAP59/FAP64 outer primers [45] and FAP085-FAP6319R inner primers [50]. By applying this method, the detection limit of CUT and FAP primer systems was shown to be 10 copies of cloned HPV10 and HPV20, respectively. Although the CUT primers were able to identify a lower number of genotypes than FAP primers, they were more efficient in detecting novel Beta and Gamma putative HPV types. Therefore, the CUT primer system could be applied for the analysis of previously characterized samples, as an additional tool to complete to whole picture of circulating PVs among humans [50,51]. These findings clearly indicate that the identification of new HPV types depends not only on the use of a highly sensitive technique, but also on the combination of different generic primer pairs.

Another approach to improve the sensitivity of systems in Beta and Gamma HPV detection is Rolling Circle Amplification (RCA). This is a mechanism employed by some viruses to multiply their circular genomes, and it has been applied in the amplification of circular plasmid vectors used in cloning. The RCA protocol uses random hexamer primers to amplify the complete genomes of PVs without knowing their DNA sequences beforehand. The multiple-primed RCA method can be used to discover previously unknown PVs, and also other circular DNA viruses. The polymerization process is primed by exonuclease-resistant random hexamers that bind onto the circular DNA template at multiple sites, and, in doing so, they generate multiple replication forks [52]. This method was employed on oral samples obtained from healthy individuals, and identified four novel HPV types, all belonging to the genus Gamma [53]. By pooling RCA reactions for subsequent consensus PCR (nested PGMY/GP5+/6+ PCR assay, FAP, CP, and broad spectrum), a higher sensitivity was obtained. However, RCA requires the presence of high HPV copy numbers in the sample to succeed in the identification of full-length HPV genomes [54,55].

Other investigators developed a novel method combining a multiplex PCR based on E7-specific primers for Beta and Gamma HPVs with an array primer extension (APEX) for typing [56,57]. Through this approach, it was possible to detect all known Beta-HPV genotypes with a sensitivity of 10 copies/reaction.

#### 2.2.3. Known and Novel HPVs

The majority of the HPV testing methods used in current research and/or clinical settings are designed to identify a restricted group of HPVs. Recently, new technologies have become available that can be used to discover novel HPV types. In this context, Next Generation sequencing (NGS) can also be used as a high-throughput assay for HPV genotyping [58]. NGS involves library preparation, product purification, quantitation and normalization steps, followed by sequencing. In most of the studies on Alpha, Beta, and Gamma HPVs, NGS uses primers that are already employed in traditional PCR assays, such as the above-mentioned PGMY and FAP [59,60]. A new recent method, named TypeSeq, is able to detect 51 Alpha-HPV types [61]. This method uses three PCR steps to obtain both target enrichment and library preparation: (1) HPV-DNA is amplified by type-specific primers; (2) universal primers amplify HPV amplicons generated in the first step; (3) sequencing adapter and barcodes are incorporated in amplicons. The sensitivity of this method ranged from 10 to 25 genome-equivalent copies. Even though the TypeSeq approach has been applied on cervical specimens, it is a promising protocol also for oral specimens. Importantly, this method demonstrates how type-specific multiplex-priming could improve the multiple type sensitivity in NGS more than a consensus priming, as previously suggested [62].

To overcome the problem of sensitivity and specificity linked to consensus primer homology, a metagenomic approach could be chosen. For example, Pastrana et al. combined the RCA method with NGS technology to improve PV detection [63]. Viral DNA was extracted from gradient fractions, amplified by random-primed RCA, and sequenced on the Illumina MiSeq Platform. In this way, 83 novel HPV genomes were discovered. A non-selective approach that theoretically can identify all HPV types has been recently described [64]. This is based on DNA fragmentation and shotgun metagenomic library preparation followed by sequencing.

Notably, the aspecific sequences/reads represent a problem for NGS determination: one milliliter of saliva might contain 10–100 million microbes and their presence is thus relevant when gargle or saliva samples are used for HPV testing. Consequently, the extracted DNA might be mostly bacterial DNA, masking the few copies of HPV-DNA contained in the samples. The considerable amount of human DNA and RNA can also reduce the reads that actually represent viral genome. Proficiency testing is required in order to assess the reliability of different NGS methods used for HPV detection.

## 3. Oral Infection by Alpha, Beta and Gamma HPVs: The Epidemiological Aspects

**Figure 2:**
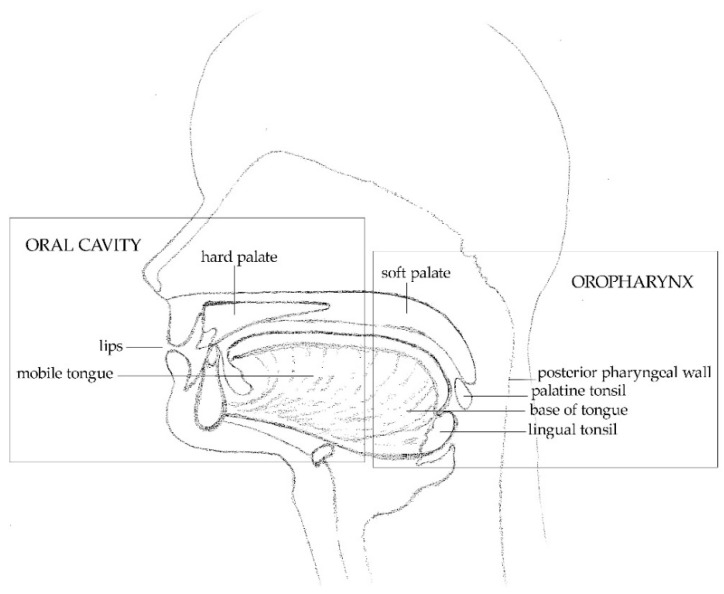
Overview of the oral cavity and oropharynx anatomy. The regions included in the two head and neck subsites that may be targeted by HPV are shown.

From a clinical point of view, the most relevant site of HPV infection at the level of the head and neck is represented by the oropharynx. However, HPV may also infect the oral cavity (Figure 2).

Certain types of samples have been proven to be the most suitable for the purposes of epidemiological studies. However, these samples are often not site-specific, as in the case of oral rinse-and-gargles. This means that the precise site of HPV infection within the head and neck region cannot be established. Even using site-specific sampling techniques, e.g., directly brushing or swabbing the specific head and neck site, contamination by HPV-positive cells possibly deriving from the contiguous areas cannot be excluded. Therefore, it is not entirely correct to define the HPV infection as “oral”, especially when using non site-specific samples, since the term “oral” solely, and specifically, refers to the oral cavity. Notwithstanding this clarification, the expression “oral” HPV infection will be used hereafter for simplicity.

**Figure 3:**
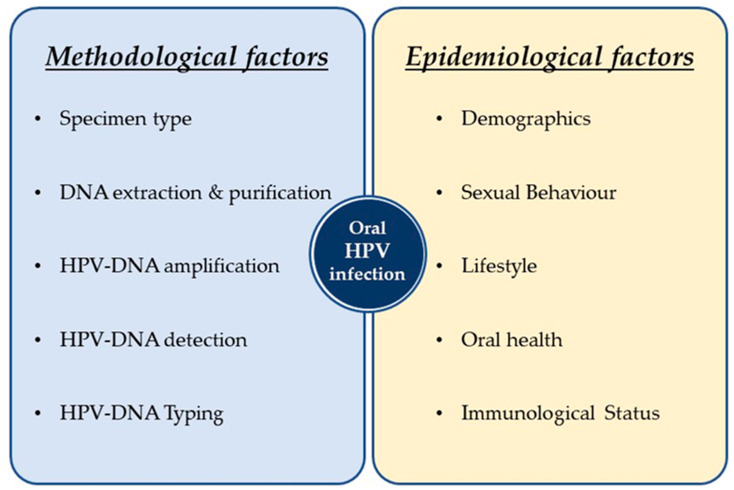
The main factors influencing the estimation of oral HPV infection. The assessment of oral HPV infection (in terms of prevalence, incidence and clearance) may be largely influenced by methods used for oral sample collection, DNA extraction, HPV-DNA amplification, detection and typing as well as by demographical, behavioral and clinical factors.

The majority of the epidemiological studies on oral HPV infection have analysed Alpha-HPVs, rather than Beta or Gamma genera. Therefore, limited data on the prevalence and natural history of the latter, as well as their determinants and potential transmission routes, have been collected. Regardless of the genus investigated, it must be highlighted that oral HPV prevalence, incidence and clearance are largely influenced by the characteristics of the study population (e.g., age, sex, ethnicity, behavior) and also by the methods used for oral sample collection, processing, and HPV detection (Figure 3).

In the following paragraphs, we will focus on oral infection by Alpha, Beta and Gamma HPVs in “healthy”, i.e., cancer-free, subjects. Other reviews can be consulted for comprehensive data regarding oral HPV in HNSCC patients [65,66].

Firstly, the epidemiology of oral HPV in the general population, at average risk of infection, will be addressed. Subsequently, this will be described for men who have sex with men (MSM) and HIV-infected individuals, which are populations at increased risk for oral HPV infection. Data about the prevalence and corresponding predictors will be reported in more depth, and a brief overview of the natural history and the risk factors associated with incidence and clearance will be also provided. Taylor et al. more comprehensively reviewed the incidence, clearance and persistence of oral HPV infections [67].

### 3.1. General Population

#### 3.1.1. Prevalence

Oral infection by Alpha-HPVs is uncommon in the general population, so a thorough analysis of its epidemiology and natural history is challenging due to the large number of individuals that need to be enrolled. A systemic review that included 18 studies conducted between 1997 and 2009 reported a prevalence of any of the Alpha-HPVs among 4581 healthy individuals below 5% [68]. Prevalence of High-risk HPVs was about 4%, and that of HPV16 1.3%. It is noteworthy that HPV16 accounted for 28% of all the oral HPV infections. Subsequently, a multinational study conducted on 1688 healthy men from the USA, Mexico and Brazil (HPV Infection in Men, HIM study) revealed a prevalence of 1.3% for High-risk HPV types, with HPV16 as the most common High-risk type in each country [69]. The oral prevalence of Alpha-HPV types among the U.S. general population (around 5600 individuals enrolled) was investigated in a cross-sectional study part of the National Health and Nutrition Examination Survey (NHANES), which reported a prevalence of 6.9% for any HPV and around 1% for HPV16, corresponding to about 2 million HPV16-infected people in the U.S. [70]. A lower prevalence (2.5% for any type and 0.4% for HPV16) was observed in a Chinese population of 1426 individuals [71]. Interestingly, oral prevalence of Beta and Gamma HPVs seems to be higher than that of Alpha-HPVs. The Chinese study found that Beta-HPVs were the most frequent cutaneous types, with a prevalence of 11.9%, compared to Gamma-HPVs, which displayed a prevalence of 2.9% [71]. The prevalence estimated for Beta-HPVs is substantially lower than that reported by others. The HIM study revealed Beta types in 29.3% of the samples collected from HIV-negative and mostly heterosexual men [6]. This estimate is even higher in another U.S. study on HIV-negative men, which detected Beta-HPVs in 74% of the participants [20]. In contrast to the relatively high frequency of Beta-HPV detection, a lower prevalence has been consistently reported for Gamma-HPVs. In fact, Bottalico et al. found these genotypes in only 12% of their oral samples [20].

#### 3.1.2. Predictors

Predictors, or determinants, of oral HPV infection have been more extensively investigated in the context of Alpha-HPVs, whereas much less is known regarding Beta and Gamma types. The most relevant predictors for the oral infection by the three different genera will be reviewed.

(i) Age. Conflicting data concerning the prevalence of Alpha-HPVs according to age have been reported. In their study on the U.S. general population, Gillison et al. highlighted a bimodal pattern, with a first peak in 30–34 year-old individuals, and a second peak in 60–64 year-old individuals [70]. Similarly, a Chinese study reported the highest prevalence of Alpha-HPVs in individuals under the age of 35, while also evidencing a marked decrease in older individuals [72]. These results contrast with those of other investigators, who revealed a higher prevalence in older subjects [71,73], which may depend on a higher incidence (resulting from changes in sexual behavior at older age, and/or a lower immune system efficiency) or a longer persistence, as reported for cervical infection in women [74,75].

Some evidence revealed older age as significantly associated with an increased detection of Beta-HPV types in the oral cavity of heterosexual males [6,71,76].

(ii) Gender. Gillison’s study revealed that any Alpha-HPV prevalence among men was almost three times higher than in women (10.1% vs. 3.6%) [70]. The substantially greater prevalence of Alpha-HPVs among men compared to women was subsequently confirmed in two consecutive NHANES cycles, being 6.6% and 1.5%, respectively, [77]. This difference has been also evidenced for HPV16, which showed a prevalence of 1.9% in men and 0.3% in women in a study that included over 13,000 people [78]. These findings might be explained by differences in the primary site of HPV infection and consequent immune response in women vs. men. In women, cervical squamo-columnar transition epithelium is a weak and trauma prone zone, where the basal epithelial cells are easily exposed to HPV infection. Differently, the keratinized epithelium of the penis is more resistant to trauma and HPV infection. Moreover, the higher and more effective immune surveillance in the mucosal epithelium of the cervix in comparison with the keratinized epithelium of the penis likely determines a more robust immune response against HPV in women. The hypothesis is that, upon acquiring a cervical infection, women develop an HPV-specific immunity that provides them with an advantage in fighting the infection when it occurs at another site, for instance at the oral cavity [77,79,80]. The significantly higher seroprevalence of antibodies against HPV16 and 18 found in women than men by Windon et al. in their cross-sectional analysis confirms the hypothesis that men are less likely to mount a detectable humoral immune response [81]. Together with the differences in immune response against HPV, sexual behavior certainly represents a relevant contributor to the higher prevalence of oral infection among men. Firstly, the risk of acquiring an oral HPV infection by performing oral sex on a woman is higher compared to that associated with the same practice on a man [82]. Furthermore, a higher number of lifetime oral sex partners was found to be significantly associated with the higher HPV16 prevalence in men [83]. Finally, the risk of acquiring oral High-risk HPVs increased with recent oral sex and the number of recent oral sex partners only among men [84].

Cutaneous HPVs also seem to be more abundant in oral samples from men than in those from women. Indeed, a prevalence of 29.3% for Beta-HPVs was found in men of HIM study [6], whereas they were detected in only 18.6% of healthy Latin American women [85]. Wong et al. confirmed the higher prevalence of Beta-HPVs in men (14.4%) compared to women (9.5%) [71].

(iii) Sexual behavior. Sexual behavior represents a major contributor to the transmission of Alpha-HPVs at oral level, although other transmission modalities are possible. Briefly, vertical transmission seems to be possible, given that the same genotypes found in the genital tract of mothers have been detected in the oral cavity of their children [86,87]. Self-inoculation has to be regarded as an additional modality of acquisition of oral HPV among women. Indeed, a meta-analysis performed on women with cervical and oral HPV infections demonstrated that the prevalence of type-concordance between cervical and oral sites was 27% [88].

The type of sexual intercourse (any sex, oral sex, vaginal sex), partnership, as well as the number of lifetime and recent partners are among the variables most frequently investigated. Gillison et al. showed that Alpha-HPV prevalence tends to increase significantly with the number of sexual partners [70]. Interestingly, it has been observed a significant increase in the prevalence of High-risk HPVs with the number of lifetime sexual partners in both sexes, with a consistently higher increase in men than women [77]. Moreover, lifetime and/or recent number of oral sex partners significantly contributes to the increase in Alpha-HPV prevalence in both women and men [71,80,85,89]. The fact that the use of barriers during oro-genital sex (condom, dental dam and plastic wraps) significantly reduces the prevalence of HPV16 and/or 18 infections corroborates the role of oro-genital contacts in the acquisition of oral HPV infection [90].

Notwithstanding the central role of oral sex, open-mouthed kissing may also contribute to oral HPV infection. Significant associations of Alpha-HPV prevalence with lifetime [89,91] and recent number of kissing partners [80] have been reported, highlighting another route of transmission.

Similarly to Alpha-HPVs, Beta and Gamma types could also be sexually transmitted, although data in this regard are inconsistent. Analysing 21 stable heterosexual couples, Moscicki and colleagues demonstrated a concordance of 27% and 20% for Beta and Gamma HPVs, respectively, more than that observed in the non-couples (10% and 4% for the two genera, respectively) [92]. Others observed an association between the greater number of lifetime oral sex partners and Beta-HPV oral infection in women [93]. Differently from the evidence provided by the previous studies, Wong et al. observed no association of oral sex and lifetime number of sexual partners with Beta or Gamma HPVs, suggesting that the transmission route for these genera could differ, at least in part, from Alpha-HPVs [71].

(iv) Smoking. Among the behavioral factors associated with oral HPV infection, the role of smoking has been debated. In the study by Wong et al., tobacco use emerged as a predictor of Alpha-HPV infection [71]. High-risk HPV prevalence, especially that of HPV16, is also higher in current smokers [78,90]. In this regard, it has been hypothesized that smoking could promote a local inflammation of the oral mucosa, as well as suppress humoral immunity through its oxidative chemical components [94]. Interestingly, while some authors observed that the association between Alpha-HPV oral infection and smoking seems to be more pronounced in women [95], others demonstrated an association in men [69]. Importantly, among men, smoking has been found to be associated with a longer persistence [96], a finding that may contribute to explaining the higher prevalence of oral HPV infection in men who are current smokers. On the other hand, an inverse association between smoking and oropharyngeal infection has been highlighted in a relatively recent study [97]. These results may be explained in part by the fact that smoking increases the level of secretory leukocyte protease inhibitor (SLPI), a protein involved in innate immunity that may, in turn, limit oral HPV acquisition [98]. It may also be hypothesized that smoking acts as a protective factor against oral HPV infection by causing a thickening of the oral epithelium. Nonetheless, this aspect needs to be further investigated. Interestingly, it has also been observed that the detection of Beta-HPVs in the oral mucosa of current smokers is much lower than that of non-smokers, although the reason behind this phenomenon remains unclear [6].

(v) Oral health. A large cross-sectional study, comprising more than 3000 participants that analysed four different measures of oral health has defined self-rated, poor-to-fair oral hygiene as an independent risk factor for oral HPV prevalence, irrespective of smoking and receptive oral sex intercourses [99]. Importantly, poor oral hygiene has been found to be associated with a higher prevalence of High-risk HPVs in the oral cavity [100]. Specifically, this cross-sectional analysis revealed that a higher approximal plaque index (API), a higher gingival bleeding index (GBI) and a greater number of extracted teeth are all significantly associated with the detection of High-risk HPVs in oral samples. In addition, the presence of Low-risk HPVs also correlated with a higher value of API and GBI [100]. These studies seem to suggest that oral health may be key for the prevention of oral HPV infection and related diseases.

#### 3.1.3. Natural History

Data on the natural history of Alpha-HPVs in the general population mainly derive from the HIM cohort. Over a 12-month period, Kreimer et al. observed incident oral infections in less than 5% and less than 1% of men for any HPV and HPV16, respectively, [94]. Median duration of infection for any HPV and HPV16 was 6.9 and 7.3 months, respectively. A subsequent study on a subset of the HIM cohort revealed that HPV16 infections that were already present at baseline (prevalent infections) were more likely to persist than HPV16 incident infections [101]. In a more recent study on over 3000 HIM participants, 10–30% of oral High-risk HPV infections were found to persist beyond 24 months (18% in the case of HPV16) [102]. A very recent study conducted in Australia showed that around one quarter of the individuals that were HPV-negative at baseline acquired new Alpha-HPV infections over a 24-month period [25].

Smoking appeared to be significantly associated with new oral High-risk HPV infections [94]. Importantly, an increase in any High-risk HPV [102] and HPV16 [101] persistence with age was observed, possibly explaining the higher prevalence of oral HPV in older individuals.

A prospective population-based study performed in Hong Kong examined the persistence and clearance of Alpha, Beta and Gamma HPV oral infection among 458 individuals that were followed up for 24 months [103]. A higher persistence and a lower clearance of Alpha-HPV infection compared to Beta/Gamma HPV infection were revealed, with the differences achieving statistical significance at 12 months (persistence rates of 22.7% vs. 9.2%; clearance rates of 31.8% vs. 45.1%). Many of the High-risk HPVs, along with several Beta/Gamma types (e.g., HPVs 21, 22, 23, 24, 98) showed persistent infections at 12 months, while others were still persisting at 24 months (e.g., HPVs 9 and 75). Interestingly, Beta/Gamma HPV persistence was found to be positively associated with the male gender, smoking and older age.

Further longitudinal studies, especially on Beta and Gamma HPVs, are needed in order to elucidate the natural history of oral HPV infection, which is still marginally understood. Findings from prospective studies may also be key to the underestimation of the role of certain HPV types in the etiology of HNSCC.

### 3.2. MSM and HIV-Infected Individuals

#### 3.2.1. Prevalence

Quite a large amount of data is available regarding oral HPV infection among MSM. They typically harbor a 3 to 5-fold higher prevalence of Alpha-HPV types compared to the general population, mainly because of their sexual behavior (e.g., frequent practice of receptive oral sex, inconsistent use of condoms during receptive oral sex, high number of sexual partners). Alpha-HPVs are even more commonly detected in MSM with a concomitant HIV infection. It has been consolidated in the evidence that HIV-infected individuals have a higher prevalence, incidence and persistence of Alpha-HPV types both in oral and ano-genital sites [3,20,104,105]. In their meta-analysis, King and colleagues analysed data from 1329 HIV-uninfected and 1886 HIV-infected MSM, reporting a pooled prevalence of 17.1% and 28.9% for any Alpha-HPV, respectively, together with a pooled prevalence of 9.1% and 16.5% for High-risk HPVs, respectively, [106]. HPV16 oral infection was also more frequent among HIV-infected MSM, being 4.7% vs. 3% in the HIV-uninfected counterparts. While HPV16 represents the most frequent genotype in oral infections, type-specific distribution in HIV-infected and HIV-uninfected subjects may vary [107,108,109]. A wider spectrum of HPVs is usually detected in HIV-infected subjects [107,109], and multiple infections are more frequent among persons living with HIV [109]. Notably, prevalence of Alpha-HPVs in HIV-infected subjects has been shown to increase with decreasing CD4+ T cell counts [110].

In agreement with the findings already outlined above for the general population, Beta and Gamma HPV types are more abundant than Alpha types also in the oral cavity of HIV-infected and HIV-uninfected MSM [111]. More specifically, this study, which was carried out using a Luminex platform, found Beta-HPVs as the most abundant genus in oral samples, being present in 53.8% of HIV-infected and in 50.3% of HIV-uninfected subjects. In particular, HPV5 was the most prevalent Beta type, regardless of the HIV status. Gamma-HPVs were less common than Beta types but still more abundant than mucosal types, and they were detected in 30.8% and 25.9% of HIV-infected and HIV-uninfected MSM, respectively. It is worth noting that about 90% of the HIV-infected MSM included in the study were undergoing successful anti-retroviral therapy, indicating that Beta and Gamma HPV oral prevalence is high also in immunorestored HIV-infected individuals. The authors also performed a NGS analysis on a subset of samples, identifying a total of 52 Beta and 75 Gamma-HPVs compared to only 16 Alpha types. Interestingly, NGS also identified putative novel Beta types, suggesting that the abundance of cutaneous types in the oral cavity is even greater than has been previously reported.

#### 3.2.2. Predictors

In the following paragraphs, the most relevant predictors of oral infection among MSM and HIV-infected individuals will be reported. Regarding Alpha-HPVs, it must be noted that the majority of the studies investigated the associations for any HPV, while a few focused on High-risk types only.

(i) Age. Age has been found to be associated with Alpha-HPV infection in MSM [91], especially those HIV-uninfected [107,112,113]. Prevalence consistently increases at older age. In the Oral/Oropharyngeal HPV in Men At Risk (OHMAR) study, their odds of being positive for Alpha-HPVs increased by 40% every 10 years of age [113]. The possible reasons for the higher prevalence in older individuals have been already outlined above. In MSM, sexual behavior may play a pivotal role in this regard, given their tendency to have new partners also at an older age [114].

Prevalence of oral infection by Beta and Gamma HPVs in MSM also tend to increase with age [113]. Explanations similar to those mentioned above may be hypothesized, although the role of sexual behavior in the acquisition of Beta and Gamma HPV infections still needs to be fully understood.

(ii) Sexual behavior. Numbers of lifetime and/or recent any sex, oral sex, rimming or tongue-kissing partners all emerged as predictors of Alpha-HPV oral infection in MSM [91,107,108,112,113]. Other variables of sexual behavior have been found to be inconsistently associated, and no evidence of association with sexual exposure has been obtained by others in HIV-infected men [115]. Practicing receptive oral sex without using a condom emerged as the major predictor of oral infection by Beta/Gamma HPVs in the HIV-infected MSM of the OHMAR study [113].

(iii) Smoking. A few studies evidenced a significant association between Alpha-HPV infection and smoking in MSM [91,107,112] or HIV-infected men [115]. Interestingly, Giuliani and colleagues found that MSM who had smoked during their lifetime displayed a significantly lower incidence of oral infection by Alpha-HPVs when compared to non-smokers [116], suggesting that smoking may act as a protective factor against oral HPV, as already discussed.

No significant association between smoking and prevalence of Beta/Gamma HPVs has been found either in HIV-infected or HIV-uninfected MSM in the OHMAR cohort [113].

(iv) Oral health. The OHMAR study investigated the role of both self-reported and clinician-rated oral hygiene and health as possible determinants of oral HPV infection in MSM. This investigation failed to observe a significant association between these factors and prevalence of Alpha-HPVs [113]. Although an independent association was not confirmed, it has been observed that poor oral health may also influence the natural history of oral HPV infection, contributing in the increased incidence of the infection by High-risk HPVs in MSM [116].

(v) HIV status. The interplay between HIV and HPV is very complex and far from clear. A recent meta-analysis defined the interaction between HPV and HIV as synergistic. Indeed, it has been demonstrated that acquisition of ano-genital HPV infection significantly increases in HIV-infected subjects [117]. In addition, it also showed that clearance of ano-genital HPV is reduced by half in cases of HIV infection. Regarding oral HPV infection, King’s meta-analysis revealed that the HIV status represents an important determinant [106], although HIV-infected individuals may harbour different levels of risk, according to their level of immunological suppression, indicated by the number of nadir and current CD4+ T-cells. Nonetheless, findings in this regard are conflicting. In a few studies, the low number of recent CD4+ T-cell count emerged as a strong predictor for Alpha-HPV infection [107,108,118,119]. A weaker immunological status also seemed to favor Beta and Gamma HPV infection, as suggested by the findings of Giuliani et al., who reported a more than 3-fold increase in the prevalence of these genera in those with a current CD4+ T-cell count <500 cells/mm^3^ [113]. However, other studies that have investigated the impact of CD4+ T-cell count on HPV oral infection failed to demonstrate a significant association with Alpha [113] or Gamma and Beta types [105].

Notably, it has been reported that the low number of recent CD4+ T-cells is a risk factor for the acquisition of Alpha-HPVs [120] and significantly reduces the clearance rate of High-risk types [116], suggesting that this parameter may also affect the natural history of oral infection by Alpha-HPVs.

The impact of anti-retroviral therapy (cART) on the epidemiological measures of oral HPV infection still needs to be dissected. Although it is clear that cART, especially currently available drug combinations, is effective in restoring the immunological status of HIV-infected individuals, its protective effect against oral HPV infection has not been demonstrated. A U.S. study found a similar prevalence of Alpha-HPVs in ever vs. never cART users [107], which was subsequently confirmed by others [115]. Consistently, a recent study that investigated the predictors of oral infection by Alpha and Beta/Gamma HPVs also evidenced no significant difference in HPV prevalence in those using vs. those not using cART for none of the genera under study [113]. Conversely, another investigation found that patients on HAART were more frequently infected by Beta or Gamma HPVs at oral level, but this study only involved 52 participants [105].

#### 3.2.3. Natural History

Several longitudinal studies investigated the natural history of Alpha-HPVs in MSM [120,121,122,123], and three additional investigations made a direct comparison of incidence and clearance in HIV-infected and HIV-uninfected MSM [116,122,124]. Incidence for Any HPV, High-risk HPVs and HPV16 tend to be higher among HIV-infected MSM [116,120,122,124], suggesting that HIV infection favors acquisition of oral HPV. A meta-analysis estimated an oral HPV incidence of 6.10/1000 person-months for the high risk population, which also included MSM [125]. This estimate was 2-fold higher than that for low risk subjects.

In terms of oral HPV clearance, some authors reported no differences between HIV-infected and HIV-uninfected MSM [122,124], while others found a lower clearance of HPV16 in the former subjects compared to the HIV-uninfected counterparts [116]. A recent study that followed-up, over 7 years, participants in two cohorts of HIV-infected and at-risk HIV-uninfected individuals found that 32% of oral HPV16 infections were persistently detected for at least 5 years [126]. Interestingly, HPV16 viral load was associated with clearance. In fact, 10-fold decrease in HPV16 copy number was associated with a 2.5-fold increase in odds of clearance.

Several factors may affect the acquisition of oral infections by Alpha-HPVs, such as the HIV status, as already mentioned. In HIV-infected MSM, it has been observed that those with more lifetime oral sex partners show an increased incidence of any Alpha-HPVs, and the risk of acquiring High-risk HPVs is independently associated with receptive oral sex practices without a condom [116]. Interestingly, a study that compared the natural history of anal and oral HPV infection in HIV-infected individuals found that the incidence of oral HPV is around 6 times lower than that of anal HPV and that the persistence is higher for anal HPV infection [127]. These findings likely contribute to explaining the higher prevalence of anal compared to oral HPV among HIV-infected subjects.

The natural history of oral infections of Beta and Gamma HPVs among MSM and HIV-infected subjects has not been explored.

## 4. Oral Infection by Alpha, Beta and Gamma HPVs: The Clinical Aspects

HNSCCs encompass malignancies located in the oral cavity, the pharynx and the larynx. These cancers are mostly attributable to the use of tobacco and alcohol. However, as already highlighted, HPV plays a significant role in the development of OPSCCs, especially those arising at the tonsils and base of the tongue. HPV role in OPSCC developing at non-lymphoepithelial subsites of the oropharynx, e.g., soft palate and the oropharyngeal walls, is less certain [128]. Compared to OPSCC, HPV contribution is less relevant in oral and laryngeal cancers, with approximately 10,000 HPV-related cases globally [10]. Instead, 42,000 cases of OPSCC are caused by HPV worldwide, but the HPV-attributable fraction largely varies according to the geographical region. The highest prevalence of HPV-attributable fraction of OPSCCs can be found in Northern Europe, Central Europe and the USA [129,130,131]. In fact, around 70% of OPSCCs in the USA are caused by HPV [132]. As also observed for the other HPV-associated cancers, HPV16 is by far the most frequent genotype in HPV-related OPSCCs [133,134,135]. Detection of HPV16 DNA in oral samples is associated with a 22-fold increased risk of developing an OPSCC [133] Compared to other High-risk types, the role of HPV16 in predicting OPSCC risk, thus seems to be unique [136].

Besides ano-genital warts, low-risk HPVs may also cause lesions at the level of the oral mucosa. For instance, HPVs 6 and 11 are the primary causative agents of the Recurrent Respiratory Papillomatosis (RRP), a chronic disease (most often with juvenile onset) characterized by recurrent exophytic papillomas in the respiratory tract [137]. Despite its mainly benign nature, there is a potential risk of malignant transformation of RRP, although rare. Other Alpha-HPVs are also involved in the development of oral lesions, such as the focal epithelial hyperplasia, a rare and benign disease that frequently arises on the lower lip, which is caused by HPV13 and 32 [138], and oral warts, where cutaneous types, such as HPV 2 and 57, have been detected [139].

Beta and Gamma HPVs have also been detected in benign lesions of the head and neck area, such as squamous cell papillomas, with a higher prevalence in those arising in the oral cavity compared to the oropharynx [140]. Notably, Gamma-HPVs have been recently found to be associated with SCC of the oral cavity and larynx [133]. However, given that many studies have focused more on Alpha-HPV involvement in HNSCC than Beta or Gamma HPVs, the percentage of HNSCC caused by Beta or Gamma types may be under-reported.

## 5. Conclusions

A large variety of methodological approaches have been used to investigate oral HPV infection by Alpha, Beta and Gamma types. New insights may come from the novelest technologies. The NGS approach, which has often been applied to cervical and anal samples, is very promising for HPV-DNA detection in oral-oropharyngeal samples, allowing the detection of a much larger HPV panel than traditional PCR methods. However, concordance between PCR and NGS is not absolute [141] and false positive results may arise from NGS utilization [59]. In fact, in oral samples, the abundant presence of human DNA or nucleic acids from bacteria or viruses other than HPV may create artifacts. In addition, cross-reactivity sometimes hampers a correct HPV typing/identification. In this regard, the standardization of methodology is clearly important to gain more knowledge regarding the natural course and impact of oral and oropharyngeal HPV infection.

HPV types of Alpha, Beta and Gamma genera are frequently detected in oral samples of cancer-free individuals, but prevalence of infection largely varies according to the population characteristics, as well as the methodological procedures used for sample collection, processing and testing. Oral infection by Beta and Gamma HPVs seems to be even more common than Alpha-HPVs, although the clinical significance of the infection caused by the former genera needs to be clarified in more depth.

Among the different head and neck sites that can be infected by HPV, the most relevant one from a clinical point of view is the oropharynx. Interestingly, HPV seems to have a specific tropism for the reticulated epithelium of the tonsillar crypts, where HPV-driven OPSCC mostly arises. Recent data suggest that this type of epithelium is not permissive for HPV infection, i.e., it cannot support the viral life cycle, making it difficult for the establishment of a productive infection. Compared to the squamous stratified epithelium of other head and neck sites, the reticulated epithelium of the tonsils may be more prone to transforming than productive HPV infections [142]. A recent study has also shown that, within the tonsils, tonsillar crypts may represent an immunosuppressive environment that facilitates HPV infection and its persistence [143].

Several factors have been described as determinants of oral HPV prevalence, incidence or clearance. Relevant predictors include demographic (age, gender), lifestyle (smoking), behavioral (sexual habits) and clinical variables (HIV status). While many of these have been extensively investigated, with either consistent or conflicting results, it is important to collect new and more solid data regarding the role of other factors, such as oral hygiene.

Although major advances have been made in the understanding of oral HPV infection, it is clearly important to better elucidate its natural history. Given HPV involvement in the development of HNSCC, this knowledge is pivotal to clarify the precise steps from infection to cancer, and to face the challenges ahead in the fight against HPV-driven tumors.

## Data Availability

Data in this review article can be used or presented for research and analysis purpose by appropriately citing the article. Any enquiry can be made with either corresponding author at her email: mariagabriella.dona@ifo.gov.it or first author at her email: eugenia.giuliani@ifo.gov.it.

## References

[1] De Sanjosé S., Diaz M., Castellsagué X., Clifford G., Bruni L., Muñoz N., Bosch F.X. (2007). Worldwide prevalence and genotype distribution of cervical human papillomavirus DNA in women with normal cytology: A meta-analysis. Lancet Infect. Dis..

[2] De Villiers E.M. (2013). Cross-roads in the classification of papillomaviruses. Virology.

[3] Donà M.G., Gheit T., Latini A., Benevolo M., Torres M., Smelov V., McKay-Chopin S., Giglio A., Cristaudo A., Zaccarelli M. (2015). Alpha, beta and gamma human papillomaviruses in the anal canal of HIV-infected and uninfected men who have sex with men. J. Infect..

[4] Forslund O., Johansson H., Madsen K.G., Kofoed K. (2013). The nasal mucosa contains a large spectrum of human papillomavirus types from the betapapillomavirus and gammapapillomavirus genera. J. Infect. Dis..

[5] Mlakar B., Kocjan B.J., Hošnjak L., Fujs Komloš K., Milošević M., Poljak M. (2014). Betapapillomaviruses in the anal canal of HIV positive and HIV negative men who have sex with men. J. Clin. Virol..

[6] Nunes E.M., Sudenga S.L., Gheit T., Tommasino M., Baggio M.L., Ferreira S., Galan L., Silva R.C., Pierce Campbell C.M., Lazcano-Ponce E. (2016). Diversity of beta-papillomavirus at anogenital and oral anatomic sites of men: The HIM study. Virology.

[7] Smelov V., Muwonge R., Sokolova O., McKay-Chopin S., Eklund C., Komyakov B., Gheit T. (2018). Beta and gamma human papillomaviruses in anal and genital sites among men: Prevalence and determinants. Sci. Rep..

[8] Altamura G., Tommasino M., Borzacchiello G. (2020). Cutaneous vs. mucosal tropism: The papillomavirus paradigm comes to an “and”. Front. Microbiol..

[9] (2012). IARC Monographs on the Evaluation of Carcinogenic Risks to Humans—Volume 100B—A Review of Human Carcinogens: Biological Agents.

[10] De Martel C., Georges D., Bray F., Ferlay J., Clifford G.M. (2020). Global burden of cancer attributable to infections in 2018: A worldwide incidence analysis. Lancet Glob. Health.

[11] Johnson D.E., Burtness B., Leemans C.R., Lui V.W.Y., Bauman J.E., Grandis J.R. (2020). Head and neck squamous cell carcinoma. Nat. Rev. Dis. Primers.

[12] Mody M.D., Rocco J.W., Yom S.S., Haddad R.I., Saba N.F. (2021). Head and neck cancer. Lancet.

[13] De Martel C., Plummer M., Vignat J., Franceschi S. (2017). Worldwide burden of cancer attributable to HPV by site, country and HPV type. Int. J. Cancer.

[14] Ramakrishnan S., Partricia S., Mathan G. (2015). Overview of high-risk HPV’s 16 and 18 infected cervical cancer: Pathogenesis to prevention. Biomed. Pharmacother..

[15] Rubin M.A., Kleter B., Zhou M., Ayala G., Cubilla A.L., Quint W.G., Pirog E.C. (2001). Detection and typing of human papillomavirus DNA in penile carcinoma: Evidence for multiple independent pathways of penile carcinogenesis. Am. J. Pathol..

[16] De Vuyst H., Clifford G.M., Nascimento M.C., Madeleine M.M., Franceschi S. (2009). Prevalence and type distribution of human papillomavirus in carcinoma and intraepithelial neoplasia of the vulva, vagina and anus: A meta-analysis. Int. J. Cancer.

[17] Doorbar J., Egawa N., Griffin H., Kranjec C., Murakami I. (2015). Human papillomavirus molecular biology and disease association. Rev. Med. Virol..

[18] Foulongne V., Sauvage V., Hebert C., Dereure O., Cheval J., Gouilh M.A., Pariente K., Segondy M., Burguière A., Manuguerra J.C. (2012). Human skin microbiota: High diversity of DNA viruses identified on the human skin by high throughput sequencing. PLoS ONE.

[19] Hampras S.S., Reed R.A., Bezalel S., Cameron M., Cherpelis B., Fenske N., Sondak V.K., Messina J., Tommasino M., Gheit T. (2016). Cutaneous human papillomavirus infection and development of subsequent squamous cell carcinoma of the skin. J. Skin Cancer.

[20] Bottalico D., Chen Z., Dunne A., Ostoloza J., McKinney S., Sun C., Schlecht N.F., Fatahzadeh M., Herrero R., Schiffman M. (2011). The oral cavity contains abundant known and novel human papillomaviruses from the betapapillomavirus and gammapapillomavirus genera. J. Infect. Dis..

[21] Muhr L.S.A., Eklund C., Dillner J. (2018). Towards quality and order in human papillomavirus research. Virology.

[22] Kellokoski J.K., Syrjanen S.M., Chang F., Yliskoski M., Syrjänen K.J. (1992). Southern blot hybridization and PCR in detection of oral human papillomavirus (HPV) infections in women with genital HPV infections. J. Oral Pathol. Med..

[23] Broccolo F., Cocuzza C.E. (2008). Automated extraction and quantitation of oncogenic HPV genotypes from cervical samples by a real-time PCR-based system. J. Virol. Methods.

[24] Seaman W.T., Andrews E., Couch M., Kojic E.M., Cu-Uvin S., Palefsky J., Deal A.M., Webster-Cyriaque J. (2010). Detection and quantitation of HPV in genital and oral tissues and fluids by real time PCR. Virol. J..

[25] Antonsson A., de Souza M., Wood Z.C., Carroll A., Van K., Paterson L., Pandeya N., Whiteman D.C. (2021). Natural history of oral HPV infection: Longitudinal analyses in prospective cohorts from australia. Int. J. Cancer.

[26] Antonsson A., Neale R.E., Boros S., Lampe G., Coman W.B., Pryor D.I., Porceddu S.V., Whiteman D.C. (2015). Human papillomavirus status and p16(INK4A) expression in patients with mucosal squamous cell carcinoma of the head and neck in queensland, australia. Cancer Epidemiol..

[27] Emmett S., Boros S., Whiteman D.C., Porceddu S.V., Panizza B.J., Antonsson A. (2018). Sexual behaviour, HPV status and p16(INK4a) expression in oropharyngeal and oral cavity squamous cell carcinomas: A case-case comparison study. J. Gen. Virol..

[28] Emmett S., Jenkins G., Boros S., Whiteman D.C., Panizza B., Antonsson A. (2017). Low prevalence of human papillomavirus in oral cavity squamous cell carcinoma in queensland, australia. ANZ J. Surg..

[29] Wendland E.M., Kops N.L., Comerlato J., Horvath J.D.C., Bessel M., Sperb D., Pimenta C., de Souza F.M.A., Mendes Pereira G.F., Falcetta F.S. (2020). STOP HPV study protocol: A nationwide case-control study of the association between oropharyngeal cancer and human papillomavirus (HPV) infection in brazil. BMJ Open.

[30] De Souza M.M.A., Hartel G., Whiteman D.C., Antonsson A. (2018). Detection of oral HPV infection—Comparison of two different specimen collection methods and two HPV detection methods. Diagn Microbiol. Infect. Dis..

[31] Fuessel Haws A.L., He Q., Rady P.L., Zhang L., Grady J., Hughes T.K., Stisser K., Konig R., Tyring S.K. (2004). Nested PCR with the PGMY09/11 and GP5(+)/6(+) primer sets improves detection of HPV-DNA in cervical samples. J. Virol. Methods.

[32] Cho H., Kishikawa T., Tokita Y., Suzuki M., Takemoto N., Hanamoto A., Fukusumi T., Yamamoto M., Fujii M., Ohno Y. (2020). Prevalence of human papillomavirus in oral gargles and tonsillar washings. Oral Oncol..

[33] Donà M.G., Pichi B., Rollo F., Benevolo M., Latini A., Laquintana V., Pellini R., Colafigli M., Frasca M., Giuliani M. (2019). Human papillomavirus detection in matched oral rinses, oropharyngeal and oral brushings of cancer-free high-risk individuals. Oral Oncol..

[34] D’Souza G., Sugar E., Ruby W., Gravitt P., Gillison M. (2005). Analysis of the effect of DNA purification on detection of human papillomavirus in oral rinse samples by PCR. J. Clin. Microbiol..

[35] Poljak M., Ostrbenk Valencak A., Gimpelj Domjanic G., Xu L., Arbyn M. (2020). Commercially available molecular tests for human papillomaviruses: A global overview. Clin. Microbiol. Infect..

[36] Manos M.M., Ting Shin Y., Wright D.K., Lewis A.I., Broker T.R., Wolinsky S.M., Manos M., Ting Y.C. (1989). The use of polymerase chain reaction amplification for the detection of genital human papillomaviruses. Cancer Cells.

[37] Jacobs M.V., de Roda Husman A.M., van den Brule A.J., Snijders P.J., Meijer C.J., Walboomers J.M. (1995). Group-specific differentiation between high- and low-risk human papillomavirus genotypes by general primer-mediated PCR and two cocktails of oligonucleotide probes. J. Clin. Microbiol..

[38] Gravitt P.E., Peyton C.L., Alessi T.Q., Wheeler C.M., Coutlée F., Hildesheim A., Schiffman M.H., Scott D.R., Apple R.J. (2000). Improved amplification of genital human papillomaviruses. J. Clin. Microbiol..

[39] Kleter B., van Doorn L.J., ter Schegget J., Schrauwen L., van Krimpen K., Burger M., ter Harmsel B., Quint W. (1998). Novel short-fragment PCR assay for highly sensitive broad-spectrum detection of anogenital human papillomaviruses. Am. J. Pathol..

[40] Kleter B., van Doorn L.J., Schrauwen L., Molijn A., Sastrowijoto S., ter Schegget J., Lindeman J., ter Harmsel B., Burger M., Quint W. (1999). Development and clinical evaluation of a highly sensitive PCR-reverse hybridization line probe assay for detection and identification of anogenital human papillomavirus. J. Clin. Microbiol..

[41] Morbini P., Dal Bello B., Alberizzi P., Mannarini L., Mevio N., Bertino G., Benazzo M. (2012). Exfoliated cells of the oral mucosa for HPV typing by SPF10 in head and neck cancer. J. Virol. Methods.

[42] Beachler D.C., Lang Kuhs K.A., Struijk L., Schussler J., Herrero R., Porras C., Hildesheim A., Cortes B., Sampson J., Quint W. (2017). The natural history of oral human papillomavirus in young Costa Rican women. Sex Transm. Dis..

[43] Bettampadi D., Sirak B.A., Fulp W.J., Abrahamsen M., Villa L.L., Lazcano-Ponce E., Salmeron J., Isaacs-Soriano K.A., Baggio M.L., Trenado M.Q. (2020). Oral HPV prevalence assessment by linear array vs. SPF10 PCR-DEIA-LiPA25 system in the HPV infection in men (HIM) study. Papillomavirus Res..

[44] Garbuglia A.R. (2014). Human papillomavirus in head and neck cancer. Cancers.

[45] Forslund O., Antonsson A., Nordin P., Stenquist B., Hansson B.G. (1999). A broad range of human papillomavirus types detected with a general PCR method suitable for analysis of cutaneous tumours and normal skin. J. Gen. Virol..

[46] Forslund O., Ly H., Reid C., Higgins G. (2003). A broad spectrum of human papillomavirus types is present in the skin of australian patients with non-melanoma skin cancers and solar keratosis. Br. J. Dermatol..

[47] Forslund O. (2007). Genetic diversity of cutaneous human papillomaviruses. J. Gen. Virol..

[48] Chen Z., Schiffman M., Herrero R., Burk R.D. (2007). Identification and characterization of two novel human papillomaviruses (HPVs) by overlapping PCR: HPV102 and HPV106. J. Gen. Virol..

[49] Vasiljevic N., Hazard K., Dillner J., Forslund O. (2008). Four novel human betapapillomaviruses of species 2 preferentially found in actinic keratosis. J. Gen. Virol..

[50] Chouhy D., Gorosito M., Sanchez A., Serra E.C., Bergero A., Fernandez Bussy R., Giri A.A. (2010). New generic primer system targeting mucosal/genital and cutaneous human papillomaviruses leads to the characterization of HPV 115, a novel beta-papillomavirus species 3. Virology.

[51] Chouhy D., Bolatti E.M., Perez G.R., Giri A.A. (2013). Analysis of the genetic diversity and phylogenetic relationships of putative human papillomavirus types. J. Gen. Virol..

[52] Rector A., Tachezy R., Van Ranst M. (2004). A sequence-independent strategy for detection and cloning of circular DNA virus genomes by using multiply primed rolling-circle amplification. J. Virol..

[53] Martin E., Dang J., Bzhalava D., Stern J., Edelstein Z.R., Koutsky L.A., Kiviat N.B., Feng Q. (2014). Characterization of three novel human papillomavirus types isolated from oral rinse samples of healthy individuals. J. Clin. Virol..

[54] Kohler A., Gottschling M., Forster J., Rowert-Huber J., Stockfleth E., Nindl I. (2010). Genomic characterization of a novel human papillomavirus (HPV-117) with a high viral load in a persisting wart. Virology.

[55] Kohler A., Gottschling M., Manning K., Lehmann M.D., Schulz E., Kruger-Corcoran D., Stockfleth E., Nindl I. (2011). Genomic characterization of ten novel cutaneous human papillomaviruses from keratotic lesions of immunosuppressed patients. J. Gen. Virol..

[56] Gheit T., Landi S., Gemignani F., Snijders P.J., Vaccarella S., Franceschi S., Canzian F., Tommasino M. (2006). Development of a sensitive and specific assay combining multiplex PCR and DNA microarray primer extension to detect high-risk mucosal human papillomavirus types. J. Clin. Microbiol..

[57] Gheit T., Billoud G., de Koning M.N., Gemignani F., Forslund O., Sylla B.S., Vaccarella S., Franceschi S., Landi S., Quint W.G. (2007). Development of a sensitive and specific multiplex PCR method combined with DNA microarray primer extension to detect betapapillomavirus types. J. Clin. Microbiol..

[58] Nilyanimit P., Chansaenroj J., Poomipak W., Praianantathavorn K., Payungporn S., Poovorawan Y. (2018). Comparison of four human papillomavirus genotyping methods: Next-generation sequencing, INNO-LiPA, electrochemical DNA chip, and nested-PCR. Ann. Lab. Med..

[59] Flores-Miramontes M.G., Torres-Reyes L.A., Alvarado-Ruiz L., Romero-Martinez S.A., Ramirez-Rodriguez V., Balderas-Pena L.M., Vallejo-Ruiz V., Pina-Sanchez P., Cortes-Gutierrez E.I., Jave-Suarez L.F. (2015). Human papillomavirus genotyping by linear array and next-generation sequencing in cervical samples from western mexico. Virol. J..

[60] Flores-Miramontes M.G., Olszewski D., Artaza-Irigaray C., Willemsen A., Bravo I.G., Vallejo-Ruiz V., Leal-Herrera Y.A., Pina-Sanchez P., Molina-Pineda A., Canton-Romero J.C. (2020). Detection of alpha, beta, gamma, and unclassified human papillomaviruses in cervical cancer samples from mexican women. Front. Cell Infect. Microbiol..

[61] Wagner S., Roberson D., Boland J., Yeager M., Cullen M., Mirabello L., Dunn S.T., Walker J., Zuna R., Schiffman M. (2019). Development of the TypeSeq assay for detection of 51 human papillomavirus genotypes by next-generation sequencing. J. Clin. Microbiol..

[62] Schmitt M., Dondog B., Waterboer T., Pawlita M., Tommasino M., Gheit T. (2010). Abundance of multiple high-risk human papillomavirus (HPV) infections found in cervical cells analyzed by use of an ultrasensitive HPV genotyping assay. J. Clin. Microbiol..

[63] Pastrana D.V., Peretti A., Welch N.L., Borgogna C., Olivero C., Badolato R., Notarangelo L.D., Gariglio M., FitzGerald P.C., McIntosh C.E. (2018). Metagenomic discovery of 83 new human papillomavirus types in patients with immunodeficiency. mSphere.

[64] Ganly I., Pei Z., Hao Y., Ma Y., Rosenthal M., Wu Z., Migliacci J., Huang B., Katabi N., Tseng W. (2021). Case control study comparing the HPV genome in patients with oral cavity squamous cell carcinoma to normal patients using metagenomic shotgun sequencing. Sci. Rep..

[65] Carlander A.F., Jakobsen K.K., Bendtsen S.K., Garset-Zamani M., Lynggaard C.D., Jensen J.S., Gronhoj C., Buchwald C.V. (2021). A contemporary systematic review on repartition of HPV-positivity in oropharyngeal cancer worldwide. Viruses.

[66] Nogues J.C., Fassas S., Mulcahy C., Zapanta P.E. (2021). Human papillomavirus-associated head and neck cancer. J. Am. Board. Fam. Med..

[67] Taylor S., Bunge E., Bakker M., Castellsague X. (2016). The incidence, clearance and persistence of non-cervical human papillomavirus infections: A systematic review of the literature. BMC Infect. Dis..

[68] Kreimer A.R., Bhatia R.K., Messeguer A.L., GonzÃlez P., Herrero R., Giuliano A.R. (2010). Oral human papillomavirus in healthy individuals: A systematic review of the literature. Sex Transm. Dis..

[69] Kreimer A.R., Villa A., Nyitray A.G., Abrahamsen M., Papenfuss M., Smith D., Hildesheim A., Villa L.L., Lazcano-Ponce E., Giuliano A.R. (2011). The epidemiology of oral HPV infection among a multinational sample of healthy men. Cancer Epidemiol. Biomark. Prev..

[70] Gillison M.L., Broutian T., Pickard R.K., Tong Z.Y., Xiao W., Kahle L., Graubard B.I., Chaturvedi A.K. (2012). Prevalence of oral HPV infection in the United States, 2009–2010. JAMA.

[71] Wong M.C.S., Vlantis A.C., Liang M., Wong P.Y., Ho W.C.S., Boon S.S., Sze R.K.H., Leung C., Chan P.K.S., Chen Z. (2018). Prevalence and epidemiologic profile of oral infection with alpha, beta, and gamma papillomaviruses in an Asian Chinese population. J. Infect. Dis..

[72] Hang D., Liu F., .Liu M., He Z., Sun M., Liu Y., Li J., Pan Y., Ning T., Guo C. (2014). Oral human papillomavirus infection and its risk factors among 5, 410 healthy adults in China, 2009–2011. Cancer Epidemiol. Biomark. Prev..

[73] Chaturvedi A.K., Graubard B.I., Pickard R.K., Xiao W., Gillison M.L. (2014). High-risk oral human papillomavirus load in the US population, national health and nutrition examination survey 2009–2010. J. Infect. Dis..

[74] Castle P.E., Schiffman M., Herrero R., Hildesheim A., Rodriguez A.C., Bratti M.C., Sherman M.E., Wacholder S., Tarone R., Burk R.D. (2005). A prospective study of age trends in cervical human papillomavirus acquisition and persistence in guanacaste, costa rica. J. Infect. Dis..

[75] Garcia-Pineres A.J., Hildesheim A., Herrero R., Trivett M., Williams M., Atmetlla I., Ramirez M., Villegas M., Schiffman M., Rodriguez A.C. (2006). Persistent human papillomavirus infection is associated with a generalized decrease in immune responsiveness in older women. Cancer Res..

[76] Pierce Campbell C.M., Gheit T., Tommasino M., Lin H.Y., Torres B.N., Messina J.L., Stoler M.H., Rollison D.E., Sirak B.A., Abrahamsen M. (2016). Cutaneous beta human papillomaviruses and the development of male external genital lesions: A case-control study nested within the HIM study. Virology.

[77] Chaturvedi A.K., Graubard B.I., Broutian T., Pickard R.K., Tong Z.Y., Xiao W., Kahle L., Gillison M.L. (2015). NHANES 2009–2012 findings: Association of sexual behaviors with higher prevalence of oral oncogenic human papillomavirus infections in U.S. men. Cancer Res..

[78] D’Souza G., McNeel T.S., Fakhry C. (2017). Understanding personal risk of oropharyngeal cancer: Risk-groups for oncogenic oral HPV infection and oropharyngeal cancer. Ann. Oncol..

[79] Beachler D.C., Jenkins G., Safaeian M., Kreimer A.R., Wentzensen N. (2016). Natural acquired immunity against subsequent genital human papillomavirus infection: A systematic review and meta-analysis. J. Infect. Dis..

[80] D’Souza G., Agrawal Y., Halpern J., Bodison S., Gillison M.L. (2009). Oral sexual behaviors associated with prevalent oral human papillomavirus infection. J. Infect. Dis..

[81] Windon M.J., Waterboer T., Hillel A.T., Chien W., Best S., Stewart C., Akst L., Troy T., Bender N., Miles B. (2019). Sex differences in HPV immunity among adults without cancer. Hum. Vaccines Immunother..

[82] Tobian A.A., Kong X., Gravitt P.E., Eaton K.P., Kigozi G., Serwadda D., Oliver A.E., Nalugoda F., Makumbi F., Chen M.Z. (2011). Male circumcision and anatomic sites of penile high-risk human papillomavirus in Rakai, Uganda. Int. J. Cancer.

[83] D’Souza G., Cullen K., Bowie J., Thorpe R., Fakhry C. (2014). Differences in oral sexual behaviors by gender, age, and race explain observed differences in prevalence of oral human papillomavirus infection. PLoS ONE.

[84] D’Souza G., Wentz A., Kluz N., Zhang Y., Sugar E., Youngfellow R.M., Guo Y., Xiao W., Gillison M.L. (2016). Sex differences in risk factors and natural history of oral human papillomavirus infection. J. Infect. Dis..

[85] Lang Kuhs K.A., Gonzalez P., Struijk L., Castro F., Hildesheim A., van Doorn L.J., Rodriguez A.C., Schiffman M., Quint W., Lowy D.R. (2013). Prevalence of and risk factors for oral human papillomavirus among young women in Costa Rica. J. Infect. Dis..

[86] Rintala M.A., Grenman S.E., Jarvenkyla M.E., Syrjanen K.J., Syrjanen S.M. (2005). High-risk types of human papillomavirus (HPV) DNA in oral and genital mucosa of infants during their first 3 years of life: Experience from the finnish HPV family study. Clin. Infect. Dis..

[87] Martinelli M., Zappa A., Bianchi S., Frati E., Colzani D., Amendola A., Tanzi E. (2012). Human papillomavirus (HPV) infection and genotype frequency in the oral mucosa of newborns in milan, italy. Clin. Microbiol. Infect..

[88] Termine N., Giovannelli L., Matranga D., Caleca M.P., Bellavia C., Perino A., Campisi G. (2011). Oral human papillomavirus infection in women with cervical HPV infection: New data from an italian cohort and a metanalysis of the literature. Oral Oncol..

[89] Pickard R.K., Xiao W., Broutian T.R., He X., Gillison M.L. (2012). The prevalence and incidence of oral human papillomavirus infection among young men and women, aged 18–30 years. Sex Transm. Dis..

[90] Gupta A., Perkins R.B., Ortega G., Feldman S., Villa A. (2019). Barrier use during oro-genital sex and oral human papillomavirus prevalence: Analysis of NHANES 2009–2014. Oral Dis..

[91] Read T.R., Hocking J.S., Vodstrcil L.A., Tabrizi S.N., McCullough M.J., Grulich A.E., Garland S.M., Bradshaw C.S., Chen M.Y., Fairley C.K. (2012). Oral human papillomavirus in men having sex with men: Risk-factors and sampling. PLoS ONE.

[92] Moscicki A.B., Ma Y., Gheit T., McKay-Chopin S., Farhat S., Widdice L.E., Tommasino M. (2017). Prevalence and transmission of beta and gamma human papillomavirus in heterosexual couples. Open Forum Infect. Dis..

[93] Winer R.L., Gheit T., Feng Q., Stern J.E., Lin J., Cherne S., Tommasino M. (2019). Prevalence and correlates of beta- and gamma-human papillomavirus detection in oral samples from mid-adult women. J. Infect. Dis..

[94] Kreimer A.R., Pierce Campbell C.M., Lin H.Y., Fulp W., Papenfuss M.R., Abrahamsen M., Hildesheim A., Villa L.L., Salmeron J.J., Lazcano-Ponce E. (2013). Incidence and clearance of oral human papillomavirus infection in men: The HIM cohort study. Lancet.

[95] Shigeishi H., Sugiyama M. (2016). Risk factors for oral human papillomavirus infection in healthy individuals: A systematic review and meta-analysis. J. Clin. Med. Res..

[96] Kero K., Rautava J., Syrjanen K., Willberg J., Grenman S., Syrjanen S. (2014). Smoking increases oral HPV persistence among men: 7-year follow-up study. Eur. J. Clin. Microbiol. Infect. Dis..

[97] Vianna L.M.S., Carneiro F.P., Amorim R., Guerra E.N.D.S., Cavalcanti Neto F.F., Tiziani V., Motoyama A.B., Bocca A.L. (2018). Oropharynx HPV status and its relation to HIV infection. PeerJ.

[98] Quabius E.S., Gorogh T., Fischer G.S., Hoffmann A.S., Gebhard M., Evert M., Beule A., Maune S., Knecht R., Ovari A. (2015). The antileukoprotease secretory leukocyte protease inhibitor (SLPI) and its role in the prevention of HPV-infections in head and neck squamous cell carcinoma. Cancer Lett..

[99] Bui T.C., Markham C.M., Ross M.W., Mullen P.D. (2013). Examining the association between oral health and oral HPV infection. Cancer Prev. Res..

[100] Dalla Torre D., Burtscher D., Solder E., Rasse M., Puelacher W. (2019). The correlation between the quality of oral hygiene and oral HPV infection in adults: A prospective cross-sectional study. Clin. Oral Investig..

[101] Pierce Campbell C.M., Kreimer A.R., Lin H.Y., Fulp W., O’Keefe M.T., Ingles D.J., Abrahamsen M., Villa L.L., Lazcano-Ponce E., Giuliano A.R. (2015). Long-term persistence of oral human papillomavirus type 16: The HPV infection in men (HIM) study. Cancer Prev. Res..

[102] Bettampadi D., Sirak B.A., Abrahamsen M.E., Reich R.R., Villa L.L., Ponce E.L., Giuliano A.R. (2020). Factors associated with persistence and clearance of high-risk oral HPV among participants in the HPV infection in men (HIM) study. Clin. Infect. Dis..

[103] Wong M.C.S., Vlantis A.C., Liang M., Wong P.Y., Ho W.C.S., Boon S.S., Leung C., Chan P.K.S., Chen Z. (2020). Persistence and clearance of oral human papillomavirus infections: A prospective population-based cohort study. J. Med. Virol..

[104] Torres M., Gheit T., McKay-Chopin S., Rodriguez C., Romero J.D., Filotico R., Donà M.G., Ortiz M., Tommasino M. (2015). Prevalence of beta and gamma human papillomaviruses in the anal canal of men who have sex with men is influenced by HIV status. J. Clin. Virol..

[105] Fatahzadeh M., Schlecht N.F., Chen Z., Bottalico D., McKinney S., Ostoloza J., Dunne A., Burk R.D. (2013). Oral human papillomavirus detection in older adults who have human immunodeficiency virus infection. Oral Surg. Oral Med. Oral Pathol. Oral Radiol..

[106] King E.M., Oomeer S., Gilson R., Copas A., Beddows S., Soldan K., Jit M., Edmunds W.J., Sonnenberg P. (2016). Oral human papillomavirus infection in men who have sex with men: A systematic review and meta-analysis. PLoS ONE.

[107] Beachler D.C., Weber K.M., Margolick J.B., Strickler H.D., Cranston R.D., Burk R.D., Wiley D.J., Minkoff H., Reddy S., Stammer E.E. (2012). Risk factors for oral HPV infection among a high prevalence population of HIV-positive and at-risk HIV-negative adults. Cancer Epidemiol. Biomark. Prev..

[108] Rollo F., Latini A., Pichi B., Colafigli M., Benevolo M., Sinopoli I., Sperduti I., Laquintana V., Fabbri G., Frasca M. (2017). Prevalence and determinants of oral infection by human papillomavirus in HIV-infected and uninfected men who have sex with men. PLoS ONE.

[109] Rollo F., Pichi B., Benevolo M., Giuliani M., Latini A., Lorenzon L., Colafigli M., Frasca M., Pellini R., Cristaudo A. (2019). Oral testing for high-risk human papillomavirus DNA and E6/E7 messenger RNA in healthy individuals at risk for oral infection. Cancer.

[110] Beachler D., D’Souza G. (2013). Oral human papillomavirus infection and head and neck cancers in HIV-infected individuals. Curr. Opin. Oncol..

[111] Gheit T., Rollo F., Brancaccio R.N., Robitaille A., Galati L., Giuliani M., Latini A., Pichi B., Benevolo M., Cuenin C. (2020). Oral infection by mucosal and cutaneous human papillomaviruses in the men who have sex with men from the OHMAR study. Viruses.

[112] Mooij S.H., Boot H.J., Speksnijder A.G., Stolte I.G., Meijer C.J., Snijders P.J., Verhagen D.W., King A.J., de Vries H.J., Quint W.G. (2013). Oral human papillomavirus infection in HIV-negative and HIV-infected MSM. AIDS.

[113] Giuliani M., Gheit T., Rollo F., Tommasino M., Latini A., Benevolo M., Pichi B., Pellini R., McKay-Chopin S., Cristaudo A. (2021). Predictors of oral infection by mucosal and cutaneous human papillomaviruses in HIV-infected and uninfected men who have sex with men of the OHMAR study. J. Clin. Med..

[114] Poynten I.M., Machalek D., Templeton D., Jin F., Hillman R., Zablotzska I., Prestage G., Holt M., Grulich A. (2016). Comparison of age-specific patterns of sexual behaviour and anal HPV prevalence in homosexual men with patterns in women. Sex Transm. Infect..

[115] Gaester K., Fonseca L.A., Luiz O., Assone T., Fontes A.S., Costa F., Duarte A.J., Casseb J. (2014). Human papillomavirus infection in oral fluids of HIV-1-positive men: Prevalence and risk factors. Sci. Rep..

[116] Giuliani M., Rollo F., Vescio M.F., Pichi B., Latini A., Benevolo M., Pellini R., Cristaudo A., Donà M.G. (2020). Oral human papillomavirus infection in HIV-infected and HIV-uninfected MSM: The OHMAR prospective cohort study. Sex Transm. Infect..

[117] Looker K.J., Ronn M.M., Brock P.M., Brisson M., Drolet M., Mayaud P., Boily M.C. (2018). Evidence of synergistic relationships between HIV and human papillomavirus (HPV): Systematic reviews and meta-analyses of longitudinal studies of HPV acquisition and clearance by HIV status, and of HIV acquisition by HPV status. J. Int. AIDS Soc..

[118] Kreimer A.R., Alberg A.J., Daniel R., Gravitt P.E., Viscidi R., Garrett E.S., Shah K.V., Gillison M.L. (2004). Oral human papillomavirus infection in adults is associated with sexual behavior and HIV serostatus. J. Infect. Dis..

[119] Robbins H.A., Fennell C.E., Gillison M., Xiao W., Guo Y., Wentz A., Kirk G.D., Mehta S.H., D’Souza G. (2015). Prevalence of and risk factors for oral human papillomavirus infection among HIV-positive and HIV-negative people who inject drugs. PLoS ONE.

[120] Beachler D.C., Sugar E.A., Margolick J.B., Weber K.M., Strickler H.D., Wiley D.J., Cranston R.D., Burk R.D., Minkoff H., Reddy S. (2015). Risk factors for acquisition and clearance of oral human papillomavirus infection among HIV-infected and HIV-uninfected adults. Am. J. Epidemiol..

[121] Darwich L., Canadas M.P., Videla S., Coll J., Molina-Lopez R.A., Cobarsi P., Sirera G., Clotet B., Can Ruti HIV-HPV Team (2014). Oral human papillomavirus type-specific infection in HIV-infected men: A prospective cohort study among men who have sex with men and heterosexual men. Clin. Microbiol. Infect..

[122] Van Aar F., Mooij S.H., van der Sande M.A., Meijer C.J., King A.J., Verhagen D.W., Heijman T., Coutinho R.A., Schim van der Loeff M.F. (2014). Twelve-month incidence and clearance of oral HPV infection in HIV-negative and HIV-infected men who have sex with men: The H2M cohort study. BMC Infect. Dis..

[123] Ong J.J., Read T.R., Vodstrcil L.A., Walker S., Chen M., Bradshaw C.S., Garland S.M., Tabrizi S.N., Cornall A., Grulich A. (2014). Detection of oral human papillomavirus in HIV-positive men who have sex with men 3 years after baseline: A follow up cross-sectional study. PLoS ONE.

[124] Mooij S.H., Boot H.J., Speksnijder A.G., Meijer C.J., King A.J., Verhagen D.W., de Vries H.J., Quint W.G., Molijn A., de Koning M.N. (2014). Six-month incidence and persistence of oral HPV infection in HIV-negative and HIV-infected men who have sex with men. PLoS ONE.

[125] Tam S., Fu S., Xu L., Krause K.J., Lairson D.R., Miao H., Sturgis E.M., Dahlstrom K.R. (2018). The epidemiology of oral human papillomavirus infection in healthy populations: A systematic review and meta-analysis. Oral Oncol..

[126] D’Souza G., Clemens G., Strickler H.D., Wiley D.J., Troy T., Struijk L., Gillison M., Fakhry C. (2020). Long-term persistence of oral HPV over 7 years of follow-up. JNCI Cancer Spectr..

[127] Beachler D.C., D’Souza G., Sugar E.A., Xiao W., Gillison M.L. (2013). Natural history of anal vs oral HPV infection in HIV-infected men and women. J. Infect. Dis..

[128] Haeggblom L., Ramqvist T., Tommasino M., Dalianis T., Nasman A. (2017). Time to change perspectives on HPV in oropharyngeal cancer. A systematic review of HPV prevalence per oropharyngeal sub-site the last 3 years. Papillomavirus Res..

[129] Anantharaman D., Abedi-Ardekani B., Beachler D.C., Gheit T., Olshan A.F., Wisniewski K., Wunsch-Filho V., Toporcov T.N., Tajara E.H., Levi J.E. (2017). Geographic heterogeneity in the prevalence of human papillomavirus in head and neck cancer. Int. J. Cancer.

[130] Castellsague X., Alemany L., Quer M., Halec G., Quiros B., Tous S., Clavero O., Alos L., Biegner T., Szafarowski T. (2016). HPV involvement in head and neck cancers: Comprehensive assessment of biomarkers in 3680 patients. J. Natl. Cancer Inst..

[131] Stjernstrom K.D., Jensen J.S., Jakobsen K.K., Gronhoj C., von Buchwald C. (2019). Current status of human papillomavirus positivity in oropharyngeal squamous cell carcinoma in Europe: A systematic review. Acta Otolaryngol..

[132] Viens L.J., Henley S.J., Watson M., Markowitz L.E., Thomas C.C., Thompson T.D., Razzaghi H., Saraiya M. (2016). Human papillomavirus-associated cancers—United States, 2008–2012. MMWR Morb. Mortal. Wkly. Rep..

[133] Agalliu I., Gapstur S., Chen Z., Wang T., Anderson R.L., Teras L., Kreimer A.R., Hayes R.B., Freedman N.D., Burk R.D. (2016). Associations of oral alpha-, beta-, and gamma-human papillomavirus types with risk of incident head and neck cancer. JAMA Oncol..

[134] Chaturvedi A.K., Engels E.A., Pfeiffer R.M., Hernandez B.Y., Xiao W., Kim E., Jiang B., Goodman M.T., Sibug-Saber M., Cozen W. (2011). Human papillomavirus and rising oropharyngeal cancer incidence in the United States. J. Clin. Oncol..

[135] Carlander A.F., Gronhoj Larsen C., Jensen D.H., Garnaes E., Kiss K., Andersen L., Olsen C.H., Franzmann M., Hogdall E., Kjaer S.K. (2017). Continuing rise in oropharyngeal cancer in a high HPV prevalence area: A danish population-based study from 2011 to 2014. Eur. J. Cancer.

[136] Fakhry C., Fung N., Tewari S.R., D’Souza G. (2020). Unique role of HPV16 in predicting oropharyngeal cancer risk more than other oncogenic oral HPV infections. Oral Oncol..

[137] Rivera G.A., Morell F. (2021). Laryngeal papillomas. Anonymous StatPearls.

[138] Vera-Iglesias E., Garcia-Arpa M., Sanchez-Caminero P., Romero-Aguilera G., Cortina de la Calle P. (2007). Focal epithelial hyperplasia. Actas Dermosifiliogr..

[139] Padayachee A. (1994). Human papillomavirus (HPV) types 2 and 57 in oral verrucae demonstrated by in situ hybridization. J. Oral Pathol. Med..

[140] Donà M.G., Pichi B., Rollo F., Gheit T., Laquintana V., Covello R., Pescarmona E., Spriano G., Pellini R., Giuliani M. (2017). Mucosal and cutaneous human papillomaviruses in head and neck squamous cell papillomas. Head Neck..

[141] Ambulos N.P., Schumaker L.M., Mathias T.J., White R., Troyer J., Wells D., Cullen K.J. (2016). Next-generation sequencing-based HPV genotyping assay validated in formalin-fixed, paraffin-embedded oropharyngeal and cervical cancer specimens. J. Biomol. Tech..

[142] Leemans C.R., Snijders P.J.F., Brakenhoff R.H. (2018). The molecular landscape of head and neck cancer. Nat. Rev. Cancer.

[143] Mattox A.K., Roelands J., Saal T.M., Cheng Y., Rinchai D., Hendrickx W., Young G.D., Diefenbach T.J., Berger A.E., Westra W.H. (2021). Myeloid cells are enriched in tonsillar crypts, providing insight into the host tropism of human papillomavirus. Am. J. Pathol..

